# Mechanotransduction assays for neural regeneration strategies: A focus on glial cells

**DOI:** 10.1063/5.0037814

**Published:** 2021-04-30

**Authors:** Nicolas Marinval, Sing Yian Chew

**Affiliations:** 1 School of Chemical and Biomedical Engineering, Nanyang Technological University, Singapore 637459; 2 Lee Kong Chian School of Medicine, Nanyang Technological University, Singapore 308232

## Abstract

Glial cells are mechanosensitive, and thus, engineered systems have taken a step forward to design mechanotransduction platforms in order to impart diverse mechanical stresses to cells. Mechanical strain encountered in the central nervous system can arise from diverse mechanisms, such as tissue reorganization, fluid flow, and axon growth, as well as pathological events including axon swelling or mechanical trauma. Biomechanical relevance of the *in vitro* mechanical testing requires to be placed in line with the physiological and mechanical changes in central nervous tissues that occur during the progression of neurodegenerative diseases. Mechanotransduction signaling utilized by glial cells and the recent approaches intended to model altered microenvironment adapted to pathological context are discussed in this review. New insights in systems merging substrate's stiffness and topography should be considered for further glial mechanotransduction studies, while testing platforms for drug discoveries promise great advancements in pharmacotherapy. Potential leads and strategies for clinical outcomes are expected to be developed following the exploration of these glial mechanosensitive signaling pathways.

## I. INTRODUCTION

Glial cells are largely involved in neural tissue remodeling throughout the physiological and pathological development of the nervous system. Glial cells also participate in the regenerative process after injury.^1,2^ These cells have the ability to perceive the mechanical signals driven by microenvironmental changes. Although neural diseases have multiple known origins (genetic defect, congenital disorder, tumor, autoimmunity, trauma, infection, environmental health, tissue mechanics, etc.), tissue mechanics is described as a major mechanism encountered and often driving pathogenesis.^3,4^ Particularly when the tissue integrity is affected, the homeostasis is dysregulated, and the mechanical changes are, therefore, among the main signal that cells are sensing. Since tissue damage or malformation leads to profound changes in the mechanical properties of the nervous tissue, it is essential to understand the response of these glial cells toward microenvironmental mechanical changes in order to restore tissue homeostasis and function. Recent discoveries concerning the mechanosensitivity of glial cells have contributed to our understanding of the mechanisms of action by which these cells probe and interact with their surrounding substrates and juxtaposed cells.

Specifically, glial cells adapt to the physiological or pathological context using mechanosensing capacity, through mechanotransduction machinery. In principle, mechanotransduction is the result of cell sensing, integration, and conversion of external mechanical cues into biochemical signals.^5^ The mechanical stimuli that are derived from cell substrate stiffness and surface tension affect the cell plasma membrane tension and result in ion influx and signaling pathways activation. On a side note, the underlying pathways (e.g., stretched-activated ion channel signaling,^6^ integrin signaling,^7^ actomyosin contractility,^8^ Hippo pathway,^9^ and the transcription factor Yap/Taz^10^) governing these mechanisms are often interconnected, depending on the nature of the mechanical signal. Thus, it is not surprising to find that glial cells are strongly involved in the pathogenesis of neurological diseases since physiological perturbations recorded in the central nervous system (CNS) distort tissue mechanical stiffness and homeostasis.^3,11^ Even slight changes in the properties of the brain extracellular matrix (ECM) or extracellular fluid pressure caused by disease progression may result in tissue stiffening and compression, which in turn lead to an alteration in the mechanical signaling. For instance, tissue stiffening is prevalent in traumatic injuries,^12^ dementia,^13^ and Alzheimer's disease (AD).^14–16^ On the other hand, soft mechanical signature of glial scars has been recorded in the CNS^17^ for multiple sclerosis (MS)^18^ and glioma.^19^

Therefore, emphasis has been placed on studying glial mechanobiology to understand the mechanotransduction signals that are involved in response to changes in microenvironment mechanical properties.^4^ The mechanobiology area has advanced in tools and techniques to reproduce as faithfully as possible the physiological constraints associated with disease development.

In this review, we emphasize the emerging focus on glial mechanotransduction with the development of biomimicking platforms to study the cell behavior in disease models through various mechanical stimuli and potential underlying findings in pharmacotherapy. Hence, we will elucidate the physiological and mechanical changes in CNS tissues that occur during the progression of neurodegenerative diseases. Then, we will discuss the current and recent advances in engineering systems that may be used to impart mechanical stresses (hydrogels, motorized forms, spatial constraints, cell-topography interaction systems, magnetic-induced traction, and micro/nanopatterning) to cells in the context of glial cells. The compilation of the latest works on mechanotransduction signaling utilized by glial cells and the recent approaches intended to model altered microenvironment adapted to pathological context by modulating substrate's stiffness and controlling cell responses will be developed. Finally, potential leads and strategies for clinical outcomes will be discussed as a perspective.

## II. MECHANICAL STIFFNESS VARIATION IN THE DISEASED AND AGING CENTRAL NERVOUS SYSTEM

Besides the well-understood involvement of biomolecular signaling in disease progression, it is becoming clear that mechanotransduction may also be involved due to changes in tissue stiffness and cytoskeletal structures.^11^ This section summarizes the variations in mechanical stiffness and ECM modifications that are encountered within the CNS and associated pathologies and encompasses the limits of current methods, highlighting the precautions and parameters to be considered when studying a particular condition. We envision that this section can be read as a database to allow the rapid establishment of a system reproducing physiological conditions that are necessary for the most reliable study of the mechanotransduction pathways used in the chosen case. Data have, therefore, been compiled in Table I enclosed, while short physiopathological description can be found in Subsections II A–II G.

**TABLE I.** Influence of the pathophysiology and measuring methods of brain stiffness in humans and animal models.

Pathology	Condition	Species	Stiffness (Pa)						Method	References
Healthy CNS	Ageing	Human	**Young adult**		**Ageing**				Magnetic resonance elastography (MRE)	27
			3.5–3.8 kPa		2.5–2.7 kPa					
		Human	3.07 kPa		2.37 kPa				MRE	14
		Mouse	25 kPa		NA				MRE	37
		Human	**Predicted stiffness at age 76**						MRE	29
			Cerebrum		2.6 ± 0.1 kPa					
			Frontal lobes		2.6 ± 0.1 kPa					
			Occipital lobes		2.8 ± 0.2 kPa					
			Parietal lobes		2.6 ± 0.2 kPa					
			Temporal lobes		2.7 ± 0.1 kPa					
			Deep GM/WM		3.0 ± 0.3 kPa					
			Cerebellum		2.2 ± 0.2 kPa					
			Sensory motor		2.8 ± 0.3 kPa					
		Human	**Predicted stiffness at age 41**						MRE	28
			Cerebrum		2.3545 ± 0.02 kPa					
			Frontal lobes		2.2326 ± 0.02 kPa					
			Occipital lobes		2.4487 ± 0.02 kPa					
			Parietal lobes		2.1414 ± 0.02 kPa					
			Temporal lobes		2.6175 ± 0.02 kPa					
			Deep GM/WM		2.2694 ± 0.02 kPa					
			Cerebellum		1.7972 ± 0.20 kPa					
			Sensory motor		2.1353 ± 0.02 kPa					
			Frontotemporal		2.4049 ± 0.02 kPa					
			composite region							
		Rat	Neonatal	Young adult	Aged				Atomic force microscopy (AFM)	59
			∼240 Pa	∼390 Pa	∼480 Pa					
	None	Bovine	White matter		Gray matter				Nanoindentation	152
			1.33 ± 0.63 kPa		0.68 ± 0.20 kPa					
		Adult Guinea pigs	Complex Young's Modulus of retinal cells						Scanning Force Miscroscope (SFM)	22
			Cell type	Force	Stiffness					
			Neurons soma	30 Hz	480 Pa					
			(Hippocampus)	200 Hz	970 Pa					
			Astrocyte soma	30 Hz	300 Pa					
			(Hippocampus)	200 Hz	520 Pa					
			Neurons soma	30 Hz	650 Pa					
			(Retina)	200 Hz	1590 Pa					
			Muller cells soma	30 Hz	260 Pa					
			(Retina)	200 Hz	600 Pa					
			Muller cells inner	30 Hz	130 Pa					
			processes	200 Hz	160 Pa					
			Muller cells outer	30 Hz	100 Pa					
			processes	200 Hz	210 Pa					
			Muller cells endfeet	30 Hz	220 Pa					
			Muller cells endfeet	200 Hz	370 Pa					
		Mouse	Converted shear modulus						Ferrule-top dynamic indentation	151
			0.5–0.8 ± 0.1 kPa							
		Cow	White matter		Gray matter				AFM	20
			1.895 ± 0.592 kPa		1.389 ± 0.289 kPa					
		Rat	All regions of the brain 150 – 300 Pa						Indentation with AFM 25-*μ*m sphere at 1 Hz and 5% strain	26
Degenerative CNS diseases	Alexander's disease	Mouse	Wild-type		Alexander				Strain- controlled rotational rheometer Hippocampus 750 *μ*m-thick brain sections	11
			446.8 ± 20.95 Pa		571.7 ± 34.74 Pa					
	Alzheimer	Human	Unit = kPa, (mean ± SD)						MRE	14
			ROI	CN	AD					
			Global	2.51 ± 0.09	2.40 ± 0.09					
			Frontal	2.65 ± 0.15	2.47 ± 0.12					
			Occipital	2.65 ± 0.13	2.68 ± 0.24					
			Parietal	2.42 ± 0.10	2.33 ± 0.10					
			Temporal	2.69 ± 0.11	2.58 ± 0.09					
			Deep GM/WM	2.79 ± 0.25	2.63 ± 0.27					
			Cerebellum	2.15 ± 0.11	2.11 ± 0.17					
			Sensory/Motor	2.82 ± 0.29	2.62 ± 0.11					
			FPT	2.63 ± 0.10	2.48 ± 0.09					
			Normal adults	Ageing	AD				Litterature-based MRE	15
			9.21 kPa	7.11 kPa	6.60 kPa					
		Mouse	Wild type651 ± 138 Pa		AD 402 ± 97 Pa				AFM Hypoxia induced in mice	36
			Wild type		AD				MRE	37
			25.0 ± 6.4 Pa		19.3 ± 3.3 Pa				
	Multiple sclerosis	Mouse	Unit = kPa, (mean ± SD)						AFM Fresh forebrain thick coronal sections Cryo-section for demyelinated tissue	33
			Fresh forebrain 1.87 ± 0.87 kPa							
				Wild-type		Demyelination				
				Remyelination						
			Corpus callosum (lysolecithin)	12.01 ± 6.16	4.34 ± 2.55	7.15 ± 0.18				
			Corpus callosum (cuprizone)	12.07 ± 3.12	8.28 ± 3.49	13.3 ± 4.90				
			Stiff lesions (MS)	NA	3.81 ± 6.73	NA				
			Soft lesions (MS)	NA	1.14 ± 1.48	NA				
		Mouse	Unit = Pa (mean ± SD)						AFM 20 *μ*m bead; k = 13 – 37 mN/m	30
				Young	Old	Hypomyelination	Demyelination			
			Cerebellum GM	260.6 ± 36.1	273.1 ± 26.9	180.5 ± 49.8		…		
			Cerebellum WM	196.7 ± 22.0	221.9 ± 36.3	239.5 ± 34.1		…		
			Cortex	253.6 ± 81.9	307.6 ± 54.8	206.1 ± 34.7		271.3 ± 17.4		
			Corpus Callosum	216.5 ± 112.5	327.7 ± 86.3	229.5 ± 21.6		139.1 ± 16.5		
			Striatum GM	286.6 ± 39.0	312.2 ± 39.5	286.7 ± 82.7		…		
			Striatum WM	315.6 ± 58.8	504.1 ± 109.0	352.2 ± 75.8		…		
			Substantia nigra	222.9 ± 51.3	278.8 ± 64.4	172.7 ± 55.2				
			pars compacta							
			Cingulum	…	…	…		312.5 ± 68.5		
		Human			Fold change				AFM Relative MS stiffness versus healthy	33
			Stiff lesions (MS)		3.81 ± 6.73					
			Soft lesions (MS)		1.14 ± 1.48					
		Human	ND						MRE	34
	Parkinson	Mouse		Control	MPTP				MRE MPTP-induced disease	25
			Hippocampal region	4.608 ± 0.719 kPa	6.958 ± 1.085 kPa					
				Entire brain	5.234 ± 0.564 kPa	6.971 ± 1.019 kPa				
Trauma	CNS injury	Rat		Uninjured	Injured				AFM indentation 1,730 measurements of two brains	17
			Cortical tissue (mean)	50–500 Pa	∼60 Pa					
			Cortical tissue (median)	285 Pa						
			Medial agranular cortex	219 ± 65 Pa						
			Lateral agranular cortex	295 ± 72 Pa						
			Anterior cingulate cortex	318 ± 75 Pa						
			Gray matter	420 Pa						
			White matter	177 Pa						
Cancer	Gioblastoma	Human	ECM stiffness	Gliotic tissue 10–180 Pa	Lower grade Glioma 50–1400 Pa		GlioblastomaBM 70–13 500 Pa		AFM ECM stiffness fresh-frozen human brain biopsies	41
			Drosophilia		Control	Induced Glioma			AFM	19
			Apparent YM	∼300 Pa	500–1500 Pa					

### A. Regional CNS stiffness variation

The CNS comprises of a heterogeneous distribution of neural cells and their respective ECM, and the organization of cellular and ECM components vary across different regions.^20–22^ This unique tissue structure confers heterogeneous cell mechanobiology and mechanical properties. The mechanical behavior of the brain and spinal cord tissues is, therefore, an essential element in understanding biological responses in the case of trauma and pathologies. Studies have revealed variation in stiffness properties emanating cellular compliance.^23^ For instance, one of the functions of glial cells is to embed the neurons, which possess higher mechanical compliance.^24^ Glial cells provide neurons with physical protection against mechanical aggression and trauma.^22^ Hence, stiffness differences have been recorded between the white and gray matter. Specifically, the white matter contains bundled myelinated axons and is often presented with stiffer properties than the gray matter, where neuron somas are found. Therefore, the cell-type dependent intrinsic stiffness variation is thought to be involved in such a phenomenon. Rheological studies have described the CNS tissue characteristics similar to those of a non-linear viscoelastic material.^24^ Indeed, neural cytoskeleton and the ECM networks stiffen when they are increasingly deformed.^24^ Also, modifying the probed axis of the brain yields different outcomes. To explain this variation, it was suggested that the brain mechanical properties vary depending on the orientation and methodology chosen, which results from the characteristic anisotropic structure of the brain. Moreover, local stiffness is likely to change after a traumatic injury or brain disease. In particular, brain stiffness decreases in neurodegenerative disorders that are thought to be related to an impairment in neurogenesis.^25^

### B. Aging

Over the course of time, many changes occur in the brain microenvironment, including loss of neuronal-glial cell connectivity and cell depletion accompanied by progressive alteration of the ECM. Thus, brain stiffness undergoes a continuous and linear decrease over aging, leading to brain atrophy.^26^ The inner brain is physiologically stiffer than cortical brain tissue, but the age-related brain atrophy is heterogeneous and results in regional brain softening due to the early shrinking of gray matter starting from the adolescence.^27^ The annual decline for the cerebrum stiffness was evaluated at −8 Pa per year for patients <60 years old^28^ and −11 Pa for patients >60 years old.^29^ The overall brain stiffness decline is estimated to be between −4.9 Pa and −13.6 Pa per year.^28^

Mechanical stiffnesses of the diverse brain region can be found in Table I. However, precautions are required when choosing the model which must be in line with the method used for an optimal definition of the corresponding model. Arani *et al.* utilized a mathematical model to predict the theoretical cerebrum stiffness of 2.56 ± 0.08 kPa for the age of 76 years old based on a 60–80 aged cohort,^29^ while Takamura *et al.* recently refined the model with an estimated cerebrum stiffness at 2.35 kPa at the age of 41 with a younger cohort comprising of 20–60-year-old patients.^28^ The difference in methodology could explain the softer measurement from the latter study.

### C. Demyelinating diseases—Multiple sclerosis

Demyelinating diseases result in a lack of myelin and are often associated with ECM modification.^13,18,30^ A particular disease associated with a local decrease in brain tissue stiffness is multiple sclerosis. The development of this inflammatory disease is characterized by the progressive destruction of myelin, leading to the loss of axonal myelination and the basement membrane. Additionally, an increase in fibrillar collagens, which results in perivascular fibrosis, is observed.^17^ In this case, the control of ECM expression by glial cells is disturbed. Subsequently, this leads to an increased proteoglycan production and hyaluronic acid (HA) secretion, which will accumulate in the vicinity of the demyelinated axon and impair remyelination.^31,32^ However, these changes in the glial microenvironment do not always follow the same process. Indeed, the tissue mechanical properties in demyelinating diseases depend on the severity and chronicity of the pathology. A recent study highlighted that acute demyelination could be reversed when followed by remyelination, resulting in reduced tissue stiffness.^33,34^ On the contrary, chronic demyelination, as in the case of multiple sclerosis, led to an increase in tissue rigidity.^33^ The mouse corpus callosum stiffness after induced demyelination was measured at 4 and 8 kPa for lysolecithin and cuprizone treatments as acute models and 16 kPa for cuprizone chronic model, respectively, while 12 kPa was recorded in the control group^33^ (Table I). Initially, the stiffness differences could be explained by the infiltration of macrophages and microglia activation in the acute lesion. Thereafter, astrogliosis was observed in the chronic lesion with glial cells expressing increased levels of cytoskeleton filaments [glial fibrillary acidic protein (GFAP) and vimentin] as well as ECM components (fibronectin, fibrillar collagen, biglycan, and decorin)^35^ in greater amount presenting the hallmark of an active lesion.

### D. Alzheimer's disease

In dementia, and particularly in Alzheimer's disease (AD), the changes in ECM composition are associated with a loss of matrix molecules' content that are essential in sustaining progenitors and stem cell niches in the brain. The brain mechanical stiffness was found to be reduced mostly in the regions that are affected by the pathology, including frontal, parietal, and temporal lobes^14,36^ (Table I). In addition, this phenomenon intensifies along with the severity of the pathology. Also, Alzheimer's disease is characterized by the formation of amyloid plaques along with intracellular neurofibrillary tangles which results in the loss of neuronal network connectivity and functionality, followed by brain atrophy.^15^ The early amyloid fibrils deposition is prone to favor an increase in brain stiffness, while the progressive synaptic loss and neurodegeneration result in an overall stiffness loss of 22.5%.^37^

### E. Spinal cord injury and CNS trauma

Glial cells are reported to be more compliant than neurons.^24,38^ However, the activation and accumulation of astrocytes and microglia in glial scar significantly increase the tissue stiffness after spinal cord injury.^22^ In acute brain injury, tenascin upregulation in sites around brain lesions stiffen the ECM,^39^ leading to long-term deleterious effects. Besides that, the remodeling of neurogenic niche after an injury can participate in long term issues in brain functionality, leading to chronic diseases.^40^ However, in a recent study, tissue softening was recorded after traumatic injury of neural tissue, which correlates with an increased expression of matrix molecules (Laminin, collagen IV) and cytoskeleton component (GFAP and vimentin) (Table I).^17^

### F. Gliosis and glioma

The modulation of the ECM expression during gliosis and glioma progression leads to an increase in brain stiffness (Table I).^3,41^ Additionally, brain stiffness was found to increase by 10 fold, reaching E = 1000 Pa in gliobastoma.^3^ As previously stated, ECM is not the only cause of brain stiffness, but glial cells also play an important role in determining tissue rigidity because of their ability to modulate their intrinsic mechanical characteristics in response to chemical or mechanical signals. For instance, ischemia-induced gliosis resulted in the upregulation of GFAP expression in Müller glial cells, which in turn led to a global cell stiffening.^42^

Considerable changes in ECM composition, cell expression, and properties occur after brain injury and in neurodegenerative diseases. Since pathological progression is associated with changes in matrix stiffness, whether it is a decrease or an increase, then, maintaining mechanical homeostasis is one of the important aspects to target and solve in order to treat a pathological state of the brain.

### G. Importance of matrix compliance throughout neurogenesis for *in vitro* models

Both ECM and intrinsic cell rigidity contribute to tissue stiffness and vary throughout neural development. During neurogenesis, neural stem cells sense the changes in their microenvironment driving the differentiation in neural lineage and following a sequence of changes. The differentiation in specific cell types is driven by preferential biomechanical cues. For instance, neuronal differentiation occurs first in brain development^43^ and neurites growth preferentially under very soft matrix stiffness conditions *in vitro* (elastic modulus E = 200 Pa), while astrocytes spread on the stiffer environment (E = 9000 Pa).^44,45^ Likewise, oligodendrocyte (OL) differentiation and maturation are triggered when the stiffness of the microenvironment is around E = 6500 Pa.^46^ Therefore, the cellular preference in the microenvironment rigidity demonstrates that an interplay occurs between intracellular contractile forces and extracellular attachment in neural cell lineage.^38^

## III. ENGINEERED METHODS TO UNDERSTAND GLIAL RESPONSE TO MECHANOTRANSDUCTION

Understanding the mechanisms of mechanotransduction (conversion of mechanical signals into a cascade of biochemical phenomena)^4^ and their contribution to development, physiology, and cerebrospinal diseases represent a major challenge in glial mechanobiology. To unravel the cellular mechanisms involved in glial mechanotransduction, engineering cell culture systems are required to better understand the molecular interplays which are specific to mechanical stimuli encountered or leading to a pathological context. Notable advances have recently been published in the field of *in vitro* engineering systems. The platforms are expected to reproduce the CNS matrix with faithful physiological conformity, thereby allowing the study of a defined signaling pathway that may be involved in the biological phenomenon that is driven by a specific mechanical stimuli. Additionally, these platforms and culture systems were designed to evaluate the biomechanical characteristics that cells could detect in order to determine the signaling pathways and downstream elements involved in various cellular behaviors, such as cell activation, proliferation, spreading, differentiation, polarization, or myelination.

The current techniques and strategies to study mechanosensing in glial cells are discussed below.

### A. Small scale techniques

Force-application techniques (AFM, optical tweezers) are standard methods to study mechanobiology and mechanical probing of mechanotransduction at the single cell level.^24,47^ AFM provide the lowest force (5–10 pN), while optical tweezers (0.1–100 pN), pipette aspiration (10 pM–1 nN), and magnetic tweezers (0.1–1 nN) can deliver higher force magnitude.^48^ These techniques remain, unfortunately, cell-selective, low throughput, and invasive methods, mimicking acute stress for cells by their possible insult to the plasma membrane and their cortical shell integrity.

### B. 2D gels

In order to modulate the external forces that cells can sense, it is necessary to adjust their microenvironment. Several approaches can be considered to generate mechanical tension such as biophysical modulation by tunable mechanical stiffness, appropriate topography, extracellular matrix coating, and culture in dynamic conditions or in three-dimensions.^49,50^ Research in natural or synthetic materials has highlighted a wide range of possibilities to obtain the desired stiffness, viscosity, or topography to study mechanotransduction (Table II). Inert and artificial substrates have been developed to offer the opportunity to study cell behavior under precisely defined mechanical conditions on a two-dimensional (2D) hydrogel.

**TABLE II.** Advantages and drawbacks of hydrogels with tunable mechanical stiffness.

Substrate	Material	Surface coating	Stiffness and specific features	Cells tested	Biological outcomes	Limits	Reference
Synthetic hydrogel	Polyacrilamide (PAA)	PDL	Stiffness 0.1–70 kPa	Rat oligodendrocyte progenitors (OPC)	Compliance: OPCs (30–150 Pa) stiffen during differentiation (OL = 40–210 Pa) independently of substratum stiffness (0.1–0.4 kPa) OPC adhesion is independent of substratum stiffness but optimal at 1 kPa OPC survival and proliferation are optimal at 0.7–1 kPa OPC migration is optimal at 0.7 kPa OPC differentiation is enhanced on stiffer substrates (1–70 kPa)	Elucidation of mechanotransduction mechanisms beyond the scope of the study Not suitable for complexes and specific morphological changes such as myelin sheath wrapping	55
		PLL Fibronectine	Stiffness 0.5–7 kPa	Mouse Oli-neu	RGD-peptide treatment increases fluid-phase endocytosis Y27632 or blebbistatin increases cell surface area Blebbistatin abolishes the RGD-peptide effect on cell area Endocytosis increases with soft matrix Rho/ROCK and myosin II inhibition by C3 transferase, Y27632 or by blebbistatin restored cell surface expansion on soft matrices Inhibition of actomyosin contractility promotes spreading of myelin-membrane sheets on a non-permissive substrate	Not suitable for complexes and specific morphological changes such as myelin sheath wrapping	46
		PDL Fibronectin Laminin	Stiffness 0.362 ± 0.065 to 9.720 ± 1.352 kPa for PAA Larger than 100 *μ*m	Rat glial precursor (CG-4) Rat neuroblastoma (B-104) Rat primary OPCs	Low substrate stiffness and merosin enhances oligodendroglial differentiation and morphological complexity Blebbistatin promotes OL differentiation on compliant substrates in presence of merosin	Only study OL differentiation in 2D environment Not suitable for complexes and specific morphological changes such as myelin sheath wrapping	7
		PDL	Stiffness 0.01–230 kPa Nonfouling and anti-adhesive; Isotropic; Elastic; Biologically inert; Homogeneity in surface topography, mechanical properties, and coating density; thin and translucent	Astrocytes	Sharp transition from the compliant to the rigid astrocyte phenotype from 1 kPa	Not suitable for complexes and topography-directed morphological study	53
		Laminin	Stiffness 0.1–75 kPa 70 *μ*m nominal thickness	Rat NSCs	ECM Stiffness biases NSC differentiation Compliant substrates yields 60% neurons, 10% astrocytes, and 5% oligodendrocytes, while stiff substrate yields 30% neurons, 20% astrocytes, and 0% oligodendrocytes On stiff ECMs, mechanotransduction inhibitors restored neuronal differentiation for all NSC populations to levels found on compliant ECMs	Not suitable for complexes and topography-directed morphological study	63
		Fibronectin	Stiffness 0.08–119 kPa	Human glioma (U373-MG, U87-MG, and U251- MG)	Mechanical rigidity regulates the motility of glioma cells through actomyosin network	Not suitable for complexes and topography-directed morphological study	57
		PDL Matrigel	100 *μ*m thick	Mouse primary spinal cord neuron	Substrate flexibility also had a significant eject on neurite branching and neural-glial differentiation	Not suitable for complexes and topography-directed morphological study	56
	Modified PAA	Laminin	Independent control on ECM peptide tethering and substrate stiffness	Rat Cortical OPC	PIEZO1 as a key mediator of OPC mechanical signaling	Not suitable for complexes and topography-directed morphological study	59
		PDL	Stiffness 0.17–3.2 kPa	Hippocampal neurons	The suppression of F-actin cytoskeleton formation improved neuritogenesis	Not suitable for complexes and topography-directed morphological study	54
Synthetic hydrogel	Polydimethylsiloxane (PDMS)	PDL	Stiffness 1.7–1700 kPa	Brain cells (neuron, glia)	Glial cells cultured on a soft substrate obviously showed a less dense and more porous actin and GFAP mesh The viscoelasticity of both neurites and glia did not show a significant dependence on the substrates' stiffness	Not suitable for complexes and topography-directed morphological study	66
		PLL	Stiffness 0.2–8 kPa	Rat primary astrocytes	Astrocytes grown on soft substrates displayed a consistently more quiescent phenotype while those on stiff substrates displayed an astrogliosis-like morphology	Not suitable for complexes and topography-directed morphological study	68
		PLL Fibronectin	Stiffness 17–173 kPa	Rat hippocampal neurons	Soft substrates provide a more optimal stiffness for hippocampal neurons	Not suitable for complexes and topography-directed morphological study	71
		Fibronectin	Stiffness 12–750 kPa	Rat NSCs	Differentiation and maturation	Not suitable for complexes and topography-directed morphological study	70
		Fibronectin Laminin	Stiffness 0.4–3.7 kPa and 750 kPa	Human NSPC (hNSPC) Rat adult hippocampal NSC (rahNSCs)	Blebbistatin abolishes spontaneous Ca^2+^ transients Substrate stiffness triggers YAP nuclear localization while Piezo1 knockdown can override the mechanical cue for localizing Yap to the nucleus Substrate stiffness reversely modulates neural differentiation (MAP-2) according to cell origin	Delicate system and does not comply to study 3D culture	6
	PAA PDMS	Sulfo-SANPAH PLL Laminin 211	0.5 kPa to 40 kPa (PAA) 4 Mpa (PDMS)	Primary rat Schwann cells (SC)	YAP/Taz remains nuclear in low cell density and relocates in the cytoplasm under blebbistatin treatment YAP/Taz is nuclear on very stiff substrate but cytopasmic on more compliant ones in presence of laminin 211. YAP/Taz nuclear localization is promoted by mechanical stretching	Not suitable for complexes and topography-directed morphological study Short term stimulation and culture	9
	PAA Fibrin	Laminin	Stiffness 0.2–9 kPa for PAA 0.25–2.1 kPa for fibrin	Rat primary neuronal and glial cells	Soft gels promotes neurites extension for neurons while astrocytes spreading is impaired with disorganized actin network	soluble factors in co-culture may hinder the mechanical stiffness effect strict observation	44
	Poly-ethylene-glycol (PEG)	Fibronectin Collagen I Collagen IV	Stiffness NC	Rat neural brain cells	Cell survival, proliferation and differentiation	Not suitable for complexes and topography-directed morphological study	74
		Poly-ornithine	Stiffness 3.4 kPa Small pores (50–150 Å)	Rat neural brain cells	Cell survival, proliferation and differentiation	Not suitable for complexes and topography-directed morphological study	73
Biological hydrogel	3D Collagen I - Hyaluronic acid	None	Stiffness 1–10 kPa	MSC	Neuronal and Glial differentiation	3D-matrix related issues (reproducibility, pore size control, difficult cell visualization and downstream analysis).	79
	3D Collagen I	None	Stiffness NC	Mouse cortical astrocyte	Cell morphology, proliferation, differentiation		107
	3D Collagen I	None	Stiffness NC	Rat primary cortical astrocyte	Proliferation and differentiation		80
	3D Collagen I	None	Stiffness NC	Human fetal cortical astrocytes	Proliferation and differentiation		81
	3D Alginate	Laminin	Stiffness 0.18–20 kPa	Rat NSCs	Cell proliferation, differentiation		85
	3D Alginate	Peptide	None	Neurons	Neurite outgrowth		107
	Agarose	Chondroitin sulfate	42.7–2006.8 dyne/cm^2^	Dorsal root ganglia	Neurite extension from DRG is influenced by the combination of mechanical barrier and ECM coating		87
	Fibrin	None	NC	Mouse spinal and cortical Neurons	ECM effect on neurite extension under compliant substrate condition		83
	3D Alginate	None	Stiffness 0.5–2.5 kPa	Rat astrocyte	Cell viability		86
	HA	Collagen IV	Stiffness 0.15–0.3 kPa	Gliobastoma	ECM effect on mechanical stiffness-induced glioblastoma proliferation		79
Synthetic and Biological Hydrogel	PAA Hyaluronan (HA)-PEGDA (Glycosyl)	Collagen I Laminin	Stiffness 0.31 ± 0.03 to 14.08 ± 1.28 kPa for PAA 0.30 ± 0.03 kPa for HA	Human Glioma (LN229 and LN18)	Glial cells can bind HA through CD44 interaction Glioblastoma cells starts to spread from 1 kPa on PAA gels but spread on lower stiffnes on HA gels in a cell line-specific fashion Laminin reduces cell spreading on HA gels	Incorporation of collagen or integrin ligand into HA crosslinked matrices can change the local stiffness by the self-assembly of fibrous structure or present epitopes otherwise seen only on stiff substrates	58
	vmIPN gel PAA-PEG	RGD RGE Laminin 1	Stiffness 0.01–10 kPa 70 *μ*m thick	Rat Adult Neural Stem Cells (NSC)	Differentiation in neuron versus glial cells Neuron 500 Pa	Not suitable for complexes and topography-directed morphological study	60
Modified biological hydrogel	Gelatin-hydroxyphenylpropionic acid	None	Stiffness 0.629–8172 kPa	Human MSC	The cells on a softer hydrogel (600 Pa) expressed more neurogenic protein markers, while cells on a stiffer hydrogel (12000 Pa) showed a higher up-regulation of myogenic protein markers	Not suitable for complexes and topography-directed morphological study	82
	Methacrylamide chitosan	Laminin	Stiffness 1–30 kPa Porous network slightly varying across stiffness	Rat NSPC	Importance of substrate stiffness in neural-glial lineage Optimal cell proliferation on 3.5 kPa surfaces Neuronal differentiation was favored on the softest surfaces <1 kPa, while OL differentiation was favored on stiffer scaffolds (>7 kPa) and astrocyte differentiation was only observed on <1 and 3.5 kPa	Not suitable for complexes and topography-directed morphological study	84

#### 1. Polyacrylamide gels

Cyto-compatible materials such as polyacrylamide (PAA) hydrogel can be fabricated with a range of elasticities (shear modulus G′ = 0.01–230 kPa) simply by varying the amount of the polymer (acrylamide) and its crosslinker (bis-acrylamide). Increasing cross-linker concentration proportionally increases the PAA gel elastic modulus until reaching an inflection point which is changed according to the PAA initial concentration.^51,52^ These modifications are not inducing an additional biological stimulus since this is a biologically inert material that does not intrinsically support cell adhesion. Thus, stiffness can be tuned without biological concern.^53^ Polyacrylamide gels exhibit a strong homogeneity in surface topography, mechanical properties, and coating density—features which are crucial for reproducibility. The high biocompatibility and the magnitude of stiffness range available makes PAA hydrogels an ideal starting matrix to reproduce CNS physiological range of mechanical stiffness. Ideally, the gel surface can be functionalized by the addition of adhesive polymers (polylysine),^53–55^ matrigel,^56^ region-specific ECM proteins (collagen I, collagen IV, laminin and fibronectin),^7,9,57–59^ or ECM peptides (RGD, IKVAV, LRE)^60,61^ to the mixture when studying particular tissue environment. ECM protein or peptide density can be tailored to define the surface chemistry of the material precisely.^62^

These models are simple to develop and can easily demonstrate cellular signaling by selectively evaluating one single effect. For instance, neuronal differentiation can be promoted by soft platforms (0.1–0.5 kPa), while stiffer ones (1–10 kPa) favored the appearance of glial cells of astrocytic phenotype.^60,63^ In the same fashion, the survival and proliferation of oligodendrocyte progenitor cells (OPC) were modulated by substrate stiffness (0.1–70 kPa).^46,55^ PAA matrices reproducing CNS mechanical properties in native and traumatic contexts (1.5 and 30 kPa, respectively) were used to highlight that stiff matrix impaired the myosin activity by inhibiting OL branching and differentiation in contrast to Schwann cells which were not affected by the change in rigidity.^64^

One must consider that one of the major disadvantages of PAA gel is the change in porosity accompanied by the change in mechanical properties, leading to modified biological responses with regard to cell fate.^65^ Furthermore, challenges and troubleshooting for PAA gels include the findings of the correct set of parameters (e.g., UV intensity, light wavelength, exposure time, distance from UV lamp, initiator concentration, gel thickness, and acrylamide and bis-acrylamide concentration), the uneven gel attachment which is a particular issue encountered during gel-gradient fabrication, and heterogenous gel thickness. Advices in methodology prior to gel manufacturing can be found in the literature.^51,52^

#### 2. Polydimethylsiloxane (PDMS) gels

Other studies used polydimethylsiloxane (PDMS) to explore mechanotransduction in neural and particularly glial cells (elastic modulus = 1 kPa–4 MPa).^9,66,67^ PDMS rigidity was tuned to study the effect of mechanical stiffness on glial proliferation, differentiation, and maturation.^9,66,68–70^ The good elastic property enables numerous applications for PDMS elastomers in micro-engineering (micropillars) or as a stretchable material to test the effects mechanical forces on cells. Details on the recent research will be specified in Sec. III E 1. However, the inert and nonfouling characteristics of these synthetic materials require additional modifications to ensure proper cell attachment and integrin signaling, including the adsorption of charge enhancers (polylysine, poly-ornithine)^66,68^ or the covalent binding of adhesive ECM proteins (laminin, fibronectin).^6,71^ The nature of the generated biological interactions and especially the coating density are critical parameters that must be tightly controlled while attempting to reproduce cell attachment model as these models may differ from the biological reality. Similarly to PAA gels, functionalization of PDMS gel with ECM protein or ECM-derived peptides can be carried out with a protein crosslinker agent. The latter are interesting for tissue engineering due to their ease of manipulation, incorporation into biomaterials and minimal impact on the mechanical characteristics of the gel.^72^ The optimal choice of the substrate-protein combination will depend on the biological relevance of the reproduced tissue microenvironment. When the complexity of the natural habitat of the cells is difficult to reproduce *in vitro* or when it is desired to decouple signaling integrins, a simplified coating model based only on the modification of the surface charge with a polylysine coating can be used to improve cell adhesion and cell spreading.^7,53^

As a result, hydrogels are often used as compliant growth substrates and can be fine-tuned to optimize their homogeneity in surface topography, mechanical properties, and coating density. For a study looking for compliant materials, it is advisable to start with PAA, while the search for a larger structural stiffness will prefer to work on a PDMS substrate.

#### 3. Other synthetic and biohybrid gels

Other synthetic gels were less frequently used to assess glial mechanotransduction by tuning their mechanical stiffness. Polyethyleneglycol (PEG) based gel of relatively low stiffness (3.4 kPa) was utilized to study neural cell biology and behavior.^73,74^ Mixing natural-based materials with synthetic compounds to enhance biophysical properties and biocompatibility of the hydrogel is a developing strategy. Bioorthogonal polymer cross-linking, such as tetrazine-norbornene ligation, can be performed to obtain *in situ* hydrogels suitable for three-dimensional (3D) cell culture.^75^ Herein, the catalytic oxidation of the dihydrogen tetrazine using horseradish peroxidase enhanced the gelation time and grant gel stiffness modulation. By applying this method, PEG was added to gelatin to form tunable stiffness composite hydrogels (storage modulus = 1.2–3.8 kPa).^76^ The viability of encapsulated cells was enhanced and could be applied to glial and neuronal cell culture as an improved method of studying mechanosensitivity in a 3D model.

#### 4. Hydrogels from natural materials

Biomaterials of natural origins have been used to culture glial cells, mainly for differentiation assays, and sometimes to assess mechanical stiffness. Among them, hyaluronic acid (HA),^58,77–79^ collagen I,^80,81^ gelatin,^82^ matrigel,^83^ fibrin,^44^ modified chitosan,^84^ alginate,^85,86^ and agarose^87^ have been used on astrocytes and neural progenitor cells (NPCs). These materials are mostly isolated from native surrounding ECM and basal lamina and contains adhesive sites for cells and, therefore, do not require additional functionalization nor surface modification to allow cell attachment. For instance, HA, which reproduces the native microenvironment that surrounds glial cells, incorporates CD-44 binding site that facilitates neural cell adhesion, while ECM polymeric proteins such as collagen, laminin, and fibronectin provides integrin binding sites. Other plant-based and non mammalian polymers would still require surface functionalization. Although substrate stiffness was not assessed for some of those hydrogels, these studies deserve to be mentioned in this section as tremendous effort in developing ECM-resembling microenvironment has been the made in the last few years. Notwithstanding, due to their natural origin, such materials suffer high variability in structure and composition with significant batch-to-batch changes in biomolecule composition and proportion. This heterogeneity added with higher structural complexity hinder proper experimental design to decouple biochemical from mechanical stimulus. For those reasons, synthetic-based hydrogels have been preferred for their bioinert properties, their well controlled content and their ease to modulate substrate stiffness. Finally, self-assembled nanopeptides have been demonstrated suitable for glial cell culture.^88^ Apparent physiological mechanical stiffness can be tuned though enzymatic addition or pH changes and hydrogel constructs have been designed to be incorporated with ECM components that are native to nerve tissues (e.g., heparan sulfate proteoglycan and laminin peptide IKVAV).^88–90^

#### 5. Recently engineered gels used to study mechanotransduction for different cell types

Recent developments in biomaterial engineering techniques have significantly improved the manufacturing capabilities of systems for analyzing cellular mechanotransduction. Hydrogels have become intensely complex and have obtained interesting new properties. Stiffness gradient hydrogel is the direct evolution from the substrate stiffness assay using various hydrogels with low to high mechanical stiffness. Gradient hydrogel can be generated by differential diffusion distance of unreacted crosslinker and monomer into pre-polymerized gel. With such system, one can attest the effect of local stiffness variation on cell mechanosensivity in a controlled manner and at a small scale.^91^

The incorporation of magnetic nano or microparticle in hydrogel permit the fabrication of a magnetic sensitive biomaterial.^92^ Magnetic hydrogels are now envisioned as a therapeutic biomaterial for spinal cord regeneration.^93^ The magnetic field allow the control of the alignment of polymer fibers to generate a topography resembling the anisotropic architecture of spinal cord microenvironment.^94,95^ Correspondingly, dorsal root ganglion neurons that were cultured in native stiffness-mimicking magnetic hydrogels demonstrated activation of mechanosensitive ion channels, TRPV4 and Piezo2.^96^ This model could be transversally applied to glial cells study.

Others have reported the assessment a novel hydrogel with rapid beating properties. Small mechanical forces are exerted through near infrared light pulse under spatiotemporal control. The authors have designed a thin, soft, and patterned synthetic gel that comprised of acrylamide variants and gold nanorods (AuNRs) for photothermal responsiveness. This approach has opened new insights into mechanotransduction studies by providing forces at low magnitudes in a natural-mimicking cyclic stimulus, rather than constant strain that is associated with classic hydrogels. For instance, this technique could be very useful in looking at quick molecular events, such as cell signaling pathway activation, nuclear translocation of mechanosensors or cell membrane dynamics.^97^

The design of hydrogels has considerably complexified to give rise to new systems with fine and intricate properties, such as photoresponsive hydrogels,^98^ thermoresponsive hydrogels,^92,99^ stiffening hydrogels,^100^ Matrix metalloproteinase (MMP)-degradable hydrogel platforms^101^ or conductive hydrogels.^102^ Among these new hydrogels, many may have properties which could satisfy new demands in the study of mechanotransduction pathways.^48,92,103–105^

### C. 3D gels

Tissue-engineered models in 3D are developing for CNS application.^80,81,106,107^ Reproducible 3D culture system based on alginate gel have been developed to monitor neurite outgrowth^108^ and to mimic astrogliosis.^86^ While tuning the material amount, mesh size evolves inversely proportional to alginate content to form the hydrogel. Alginate hydrogel has typical mechanical properties with a solid-like character, represented by storage modulus (G′), predominant over liquid-like viscous feature or loss modulus (G″). Also, PEG was used to construct a three-dimensional hydrogel and demonstrated the importance of mesh nets size along with storage modulus to modulate OPC proliferation and lineage commitment.^109^ The focus is now on developing 3D micropatterned biomaterial systems which enable the seamless integration with experimental cell mechanics in a controlled 3D microenvironment.^110^ For instance, synthetic fibrous collagen-wise material with tunable mechanics and user-defined architecture has been developed and could be applied for glial cell culture.^111^ Also, a biosynthetic elastin-like matrix was used to study neural progenitor cell (NPC) differentiation exposing cells to native brain tissue stiffness (elastic moduli ≈ 0.5–1.5 kPa).^112^

### D. Microbeads and spatial constraints

Other types of mechanical stress can be generated by the addition of micro-objects restricting the interstitial space and exerting spatial constraints on the cells (Table III). Microspheres are used in culture to generate this spatial constraint in high cell density, reproducing spatial restriction encountered in brain diseases such as gliosis or after injury and fluid infiltration leading to tissue compression. Space reduction induced by plating microspheres enhances OPCs' differentiation and generation of myelinated fibers.^113^ This method is simple and potentially useful to reproduce space constraint. However, it does not mimic the normal physiological conditions and lack the matrix substrate interaction for studying the mechanotransduction involved in other phenomenon.^114^

**TABLE III.** Advantages and drawbacks of systems using spatial constraint and magnetic particles to study glial mechanotransduction.

Substrate	Material	Surface coating	Device/Mechanical stimulation	Stiffness and specific features	Cells tested	Biological outcomes	Limits	References
Biological hydrogel	Stretched silicon sheets	Matrigel	Compression–space restriction	Stiffness non communicated (NC)	Mouse oligodendrocyte progenitor cell (OPC)	Stimulation promotes OL differentiation by heterochromatin formation through Syne1 (LINC) mechanotransduction	Comparable to microsphere space constraint	133
		Polystyrene Thick ACLAR 33 C film	Matrigel Collagen I	Custom-built mechanobioreactors with extension chamber	Stiffness NC	Rat astrocyte	Living scaffold emulating developmental conditions	Mimic radial glia	137
								Result robustness is coating-dependent	
								Required astrocyte processes network with sufficient resilience and growth capacity	
							More robust stretched processes at 12.5 *μ*m/h		
							The applied diplacement rate is different than stretched-injury models	Heterogeneous stretch within cultures with the most robust stretch seen near the corners of the towing membranes	
								Changes in astrocyte processes thickness underscore the heterogeneous effect of the mechanical tension	
	Synthetic Hydrogel and substrate	PAA PDMS	Sulfo-SANPAH PLL Laminin 211	Cell density	Stiffness 0.5 kPa to 40 kPa (PAA) 4 Mpa (PDMS)	Primary rat Schwann cells (SC)	YAP/Taz remains nuclear in low cell density and relocates in the cytoplasm under blebbistatin treatment	Not suitable for complexes and topography-directed morphological study	9
								Short term stimulation and culture	
	Biological hydrogel	Agarose	Chondroitin sulfate Laminin	Mechanical stiffness and interface hindering	Shear modulus 42.7–2006.8 dyne/cm^2^	Dorsal root ganglia (DRG)	Neurite extension from DRG is influenced by the combination of mechanical barrier and ECM coating	Not suitable for complexes and topography-directed morphological study	87
	Magnetic MNP	Superparamagnetic iron oxide nanoparticles	PLL	Mechanical tension through generation of magnetic force	Zeta potential of the PLL-SPIONs (∼+15 mV) at pH = 7.0 Saturation magnetization 351.6 kA/m	Schwann cells	Integrin-mediated migration of Schwann cell across astrocyte monolayer is enhanced by the presence of a magnetic field	Cell uptake of foreign body could alterate the signaling and behavior. The induction of magnetic field does not reproduce the nature of the forces encountered *in vivo* by the cells	141
		Superparamagnetic iron oxide nanoparticles	PLL	Mechanical tension through generation of magnetic force	Zeta potential for naked-MNPs (–20 mV) and for PLL-MNPs (+10 mV) Saturation magnetization MS = 78 Am^2^/kg	Schwann cells	Integrin-mediated migration of Schwann cell assessed by the presence of magnetic field		140

### E. Nanotopography

Topographical cues have been demonstrated to play an important role in determining cell fate. Topographical interaction can be studied by specific patterns and designed culture substrate to mimic defined conditions encountered in CNS tissues (stem cell niche, topography-directed neurogenesis, demyelination, axon and neurite extension, etc.). Different methods exist to design a particular topography (Table IV). Among them, nanotopography can be used to design precise (lithography) or random surface features (nanotube, porous membrane, electrospinning, self-assembled nanofiber) that may be applicable to study glial cell behavior when designing scaffold for neural regeneration.^115^ These systems may be extended to understand glia mechanotransduction.

**TABLE IV.** Advantages and drawbacks of microengineered scaffolds to study glial mechanotransduction.

Type	Material	Surface coating	Device/Mechanical stimulation	Stiffness and specific features	Cells tested	Biological outcomes	Limits	References
Nanograting	PDMS	Laminin	Nanotopography	Stiffness NC	Human embryonic stem cell (H1)	High actomyosin contractility induced by a nano-grating topography is crucial for neuronal maturation	Cells adhesion is restricted to the topography and limits cell spreading and migration behavior.	119
						Blebbistatin and ML-7 reduces the expression level of microtubule-associated protein 2		
	PDMS	Poly-L-ornithine Laminin	Micro- and nanotopography	Stiffness NC	Mouse primary neural progenitor	Glial differentiation is enhanced on isotropic 2 *μ* m holes and 1 *μ* m pillars in contrast to neuron differentiation which is enhanced on anisotropic gratings and isotropic 1 *μ*m pillars		117
	PDMS	Fibronectin	Nanotopography	Stiffness NC	hMSC	FAK phosphorylation was required for topography-induced neural differentiation while FAK overexpression overruled the topographical cues in determining cell lineage bias		115
Micropatterning	Fused silica	PLL	Microtopography	25 *μ*m height and 50 *μ*m diameter	Rat and mouse OPC	Hightroughput method identified a cluster of antimuscarinic compounds that enhance oligodendrocyte differentiation and remyelination		118
Micropatterning and electrospinning	Gelatin PLA	None	Suspended microfiber	Stiffness ≈20 Mpa	Human glioblastoma cells	The low apparent stiffness of the fibers is biomimetic of fibril components of the extracellular matrix, facilitating adequate cell−cell and cell−substrate interactions for the cell aggregates to remodel the fiber network		120
Electrospun artificial axons	PLA	PDL, laminin	Topography ECM interaction	ND	Rat cortical OPC	Differentiation Myelin sheath formation		122
	PCL PLA Gelatin	PDL	Topography substrate stiffness	Intrisic material stiffness Gelatin: 2–4 MPa PCL: 0.5–1 × 10^3^ Mpa PLA: 2–3 × 10^3^ MPa	Rat cortical OPC	Differentiation Myelin sheath formation	High intrisic and mechanical stiffness values	124
				Mechanical stiffness PCL: 0.014–0.050 N m^−1^				
	Polystirene	PLL	Topography	Stiffness NC	Rat cortical OPC	Differentiation Myelin sheath formation		121
3D Printing artificial axons	PDMS pHEMA poly(HDDA-co-starPEG	PDL Laminin Fibronectin	Topography	Stiffness Fibers 0.1–10 000 kPa PDMS ink E = 976 kPa pHEMA ink E = 88–333 kPa poly(HDDA-co-starPEG) ink E = 0.42–140 kPa	Rat cortical OPC	Differentiation Myelin sheath formation	Large fibers diameter	127

#### 1. Nanolithography obtained patterns

PDMS is a suitable starter biomaterial to design well-defined patterns. Customizable multi-architecture chip (MARC) array based on PDMS was used to build distinct topographies of various architectural complexities, including both isotropic and anisotropic features, in nano- to micrometer dimensions, with different aspect ratios and hierarchical structures.^116,117^ The cost-effective feature of micropillars make this method suitable for high throughput screening assays for glial cell behavior to topographical cues.^118^ This method could be effectively applied to study glial mechanotransduction in an attempt to reproduce the particular brain or spinal topography. Anisotropically grating patterned substrates are used to study glial cell differentiation.^117^ In particular, Ankam *et al.* used this technique to elucidate the underlying mechanisms of topography-induced differentiation of human embryonic stem cells (hESCs) toward neuronal lineages.^119^ In addition, suspended microfibers were recently fabricated via low-voltage 3D micropatterning.^120^ Nonetheless, the restriction of cell anchoring sites is also the main disadvantage of this technique, which considerably limits the mechanisms of cell spreading and migration that can generate signaling biases in mechanotransduction pathways.

#### 2. Artificial axons

Henceforth, growing interest in establishing the mechanosensing capacity of myelinating cells by modulating microenvironmental and biomechanical characteristics *in vitro* is arising. Although the link between mechanotransduction and myelination is not fully determined at the molecular level, the activation of the mechanotransduction pathways is thought to be essential for the quality of myelination.^38^ A focus on developing synthetic neuronal axons displaying the biochemistry, morphology, and carrying biophysical characteristics of its biological analog has been increasing over the past recent years. Electrospinning can produce nano- to microfibers mimicking the axon biophysical cues including fiber diameter (0.5–2 *μ*m), alignment and density. Therefore, electrospun artificial axons had been proposed as a model for studying myelination since the geometry of the substrate could facilitate cell surface interaction, spreading, and wrapping in the absence of neural factors.^121^ Under controlled biochemical cues of soluble factors determining their differentiation and maturation, OLs can extend their plasma membrane and generate a simulacrum of myelin sheath around the artificial axons.^122^ The direct visualization and quantification of myelin formation offered by these biomimetic platforms is thought to be an optimal system for pharmacological agent screening and testing.^123,124^ In this sense, artificial fibers can be used to assess stiffness changes in the microenvironment by modulating the nature, length and diameter of the fibers.^125^ Suspended fibers are thought to be an optimized model that overcome the influence of the support (glass generally) to better control the structural stiffness of the fiber mesh.^124^ The intrinsic and mechanical stiffness of these suspended fibers can be tuned to study mechanotransduction pathways in OL differentiation and myelination.^125^ Several biomaterials have been tested as a fiber substrate, including polystyrene, polylactic acid (PLA), polycaprolactone (PCL), and gelatin, and showed the possibility of modulating myelination in defined conditions.^122,125^ The stiffness range of such model is the closest to native condition, as compared to other topography study system (micropillars) or fiber fabrication systems.

Another aspect that compels the high interest in fiber-based myelination platform is the possibility to test out drugs and discover potential target for therapeutic treatment. Inert fibers are able to receive different types of coating to functionalize their surface in order to sustainably deliver non-viral genes (microRNA) and protein drugs.^123^ Classical myelination assays, which consists ofOL-neuron co-culture, have very low throughput and are time-consuming. Thus, this can be considered as a good developing model for high throughput screening system for drug testing to target myelination process under defined mechanical conditions.^125^ Furthermore, current platforms for high throughput *in vitro* assays have been designed to assesses myelination of living axons. This type of assay is optimal for screening large compound libraries to identify new targets and drugs that stimulate myelination.^126^ Although, this system is advantageous on non-biological substrate which does not incorporate the complex cellular neural-glial interactions, it lacks the mechanical aspect of it. Engineering systems with tunable mechanical properties could be therefore developed for co-culture study.

Although effort have been put to develop 3D culture for glia, electrospun fibers are material-dependent regarding their intrinsic stiffness. Hence, structural and intrinsic stiffnesses remain vastly far from native CNS and axon stiffness respectively.^111,125^ Nonetheless, there is no available platform to date that better mimics both topography and CNS stiffness to study the behavior of glial cells in response to stiffness changes.

Additionally, recent findings have demonstrated the fabrication of 3D-printed engineered artificial axons.^127^ The authors described their product as minimally supported aligned fibers in mechanically compliant range (0.1–1000 kPa) and with a relatively small diameter (5–20 *μ*m). The appeal of this platform lies in the fact that those features can be independently modulated to reproduce specific physio-pathological states arousing interest to study myelination process under defined conditions encountered by OLs *in vivo*. Their work showed that myelin production and wrapping is dependent on fiber diameter, stiffness, and surface ligand interaction. The application of this model to mechanical stretching platform can be considered as a challenge. Improvements are required to develop a universal and reproducible system that could be scaled up. The production of artificial fibers by 3D printing technology could be an alternative but the resolution is not able to produce axon-like diameters under 10 *μ*m at the moment. The ability to manufacture an axon-like material with a 3D printer is a promising technology that is expected to develop further in the future. Notwithstanding, 3D-printing machine definition is emerging and constantly upgrading, granting expectations to improve on the resolution matter soon.^127^

### F. Externally applied forces (motorized platform)

Advanced platforms can be compatible with dynamic systems that can add a new dimension of mechanical stress related to tissue deformation (Table V). Two types of motorized devices are found in literature, the tensile strain and stretching platforms. Dynamic cell culture systems are often used to apply mechanical stimulus to reproduce physiological constraints and forces perceived by cells. Especially in the case of glial cells, dynamic platforms can mimic cell elongation, such as axonal growth, during brain development to assess glial response to the generated forces. In addition, stretching stresses reproduce the deformation of CNS tissues following trauma.^128–130^ Thus, a compliant and flexible matrix is necessary to obtain a deformable cell substrate. For instance, elastic polymeric gels and thin crosslinked silicone films following traction or stretched force are used as culture models to measure the effect of substrate rigidity on cell mechanistics. As previously stated, the matrix elastic properties can be modified by changing the ratio of monomer to crosslinker in polyacrylamide gels.^131^ In biomechanics, the force-velocity relationship between the matrix compliance and the ability of cells to be mechanosensitive can be explained by the two-spring model based on the linear elasticity of hydrogels. In summary, soft substrates increase the force needed to maintain the stability of an adhesion, while on rigid surfaces this force is reduced when the actomyosin system is already fully mobilized to stabilize the focal adhesions.^132^ Therefore, the optimal situation for a cell would be to have a surrounding matrix stiffness of the same magnitude as that of cell compliance.

**TABLE V.** Advantages and drawbacks of motorized platforms to study glial mechanotransduction.

Substrate	Material	Surface coating	Device/Mechanical stimulation	Stiffness and specific features	Cells tested	Biological outcomes	Limits	References
Biological hydrogel	Stretched silicon sheets	Matrigel	Cell-shortening device/Compression–space restriction	Stiffness non communicated (NC)	Mouse oligodendrocyte progenitor cell (OPC)	Stimulation promotes OL differentiation by heterochromatin formation through Syne1 (LINC) mechanotransduction	Comparable to microsphere space constraint	133
	Polystyrene Thick ACLAR 33 C film	Matrigel Collagen I	Custom-built mechanobioreactors with extension chamber/Stretch-growth Long process outgrowth	Stiffness NC	Rat astrocyte	Living scaffold emulating developmental conditions	Mimic radial glia	137
						More robust stretched processes at 12.5 *μ*m/h	Result robustness is coating-dependent	
						The applied displacement rate is different than stretched-injury models	Required astrocyte processes network with sufficient resilience and growth capacity	
							Heterogeneous stretch within cultures with the most robust stretch seen near the corners of the towing membranes Changes in astrocyte processes thickness underscore the heterogeenous effect of the mechanical tension	
Synthetic hydrogel	PDMS plates fabricated from Sylgard 184 silicone	Fibonectin	Tensile strain device/10% static tensile strain for 48h	Stiffness NC	Rat primary OPC	Early differentiation in OL investigated under mechanical stimulus shows reduction in cell migration and microtubule network reorganization	Only tested tensile strain, which may not encompass the complexity of mechanical stresses encountered *in vivo*	67
		PDL Laminin Fibonectin	Tensile strain device (1) Biaxial static tensile strain of 15% for 24 h (Proliferation) (2) 10% static tensile strain for 3–5 Day (Differentiation)	Stiffness NC	Rat primary OPC	Sustained tensile strain inhibits OPC proliferation and promoted OL differentiation through chromatine reorganization and nucleus shape changes	Only tested tensile strain, which may not encompass the complexity of mechanical stresses encountered *in vivo*	134
Synthetic substrate	Teflon disk with silicon membrane	Laminin Fibronectin	Tensile Strain/10% static equibiaxial stretch	Stiffness Unstretched 10 kPa Stretched 1.6 Mpa	Mouse cortical neural stem/progenitor cell (NSPC)	Stretch impacts NSPC differentiation into OL, but not neurons or astrocytes, and is dependent on ECM-integrin linkages	The stiffness range is high and does not mimic a physiological range	135
					Rat hippocampal NSPC	Generation of OL decreased on laminin		
Synthetic substrate ?	Silicon chamber	Laminin	Cell stretching Shear stress (1) Computer-controlled stepping motor machine	Stiffness NC	Rat OPC	YAP regulates OL morphology and interactions with neuronal axons Mechanical stretching induces	Suitable only for early differentiation step but does not encompass topographical cues to study OL maturation and myelination	10
			(2) Shear stress by flask rotation					
						nuclear YAP translocation and focal adhesion assembly		
						Shear stress decreased the number of OL processes		
Synthetic Hydrogel and substrate	PAA PDMS Silicone sheets	Sulfo-SANPAH PLL Laminin 211	Cell density Substrate stiffness Uniaxial stretching	0.5 kPa to 40 kPa (PAA) 4 Mpa (PDMS)	Primary rat Schwann cells (SC)	YAP/Taz remains nuclear in low cell density and relocates in the cytoplasm under blebbistatin treatment	Not suitable for complexes and topography-directed morphological study Short term stimulation and	9
						YAP/Taz is nuclear on very stiff substrate but cytopasmic on more compliant ones in presence of laminin 211. YAP/Taz nuclear localization is promoted by mechanical stretching		
							culture	
Modified biological hydrogel	Methacrylamide chitosan	Laminin	Mach 1 micromechanical testing system/Uniaxial stress-relaxation Substrate stiffness	Stiffness 1–30 kPa	Rat NSPC	Used for mechanical testing of the gels, not assessed for cells	Not suitable for complexes and topography-directed morphological study	84
				Porous network slightly varying across stiffness				
Synthetic substrate	Bioflex Plates	Collagen I	biaxial stretch	Stiffness NC	Adult astrocytes	Mechanical stress activates stretched-activated ion channels and regulates the expression of endothelin and endothelin receptors in astrocytes	Not suitable for complexes and topography-directed morphological study	136

In practice, a Matrigel-coated pre-stretched silicon substrate was used as a matrix to directly address the effect of mechanical forces on nuclear heterochromatin organization in OPCs. The platform was mounted in a device that generates uniaxial cell deformation upon mechanical release of the substrate.^133^ Such assay resumes mechanical compression by spatial restriction and exhibits comparable stimulus generated by microsphere space constraint. Thus, the authors identified SYNE1 as a key mechanotransducer in the nuclear envelope complex (LINC) and transmits the mechanical stress to the nucleoskeleton, subsequently leading to the formation of heterochromatin, a main step in the OL differentiation. Also, the application of tensile strain to cells plated on elastomeric PDMS plates is a model developed recently.^67,134^ Mechanical stretching was used to demonstrate the differential cell commitment to glial lineage along with the importance of ECM nature to direct oligodendrocyte differentiation,^135^ or the regulation of the astrocytic endothelin secretion through ion channel activation.^136^ Glial morphological changes and nuclear translocation of mechanotransducer Yes-Associated Protein (YAP) could be verified by the dynamic stimulation.^9,10^ Katiyar *et al.* used a stretching platform coated with Matrigel or collagen I to engineer a “living scaffold” based on long astrocytes processes.^137^ Such model could open the way to explore mechanotransduction in co-culture model on motorized platforms. Also, a novel cell-stretching array platform was designed to obtain defined cellular alignment *in vitro,*^138^ which is an interesting feature that can be easily applied for glial cell culture.

Yet, these platforms still lack a long response time and are yet limited in generating homogeneous uniaxial or bidirectional forces regardless of the spatial stimulation. Recent advancement in engineering three-motorized stage system allowing imaging during the two phases of the cyclic stretch could be investigated to design multiparametric stimuli.^139^ Nonetheless, those dynamic platforms are using hydrogel-based substrates to assess material deformation. Therefore, the recommendation to wisely choose the appropriate material and stiffness range apply herein. The type and nature of polymer can modify the cell response and adhesion properties and may require additional coating to ensure proper cell anchorage and focal adhesion formation.

### G. Magnetic particle (MNP) to reproduce axon-traction force

In a different field, iron oxide (Fe_3_O_4_) magnetic nanoparticles (MNPs) are able to produce mechanical tension provoking axon elongation and growth.^140,141^ Their use was proposed to potentially improve nerve regeneration and to implement guidance for regenerating axons through cell magnetic actuation.^142^ In order to develop novel functional nanotools, the MNPs could be used as an *in vitro* system assay to promote axonal elongation/growth by exploiting the mechanical forces that act on MNP-neurons and thus study remyelination in co-culture platform with OL.

## IV. GLIAL MECHANOSENSORS

**FIG 1.**
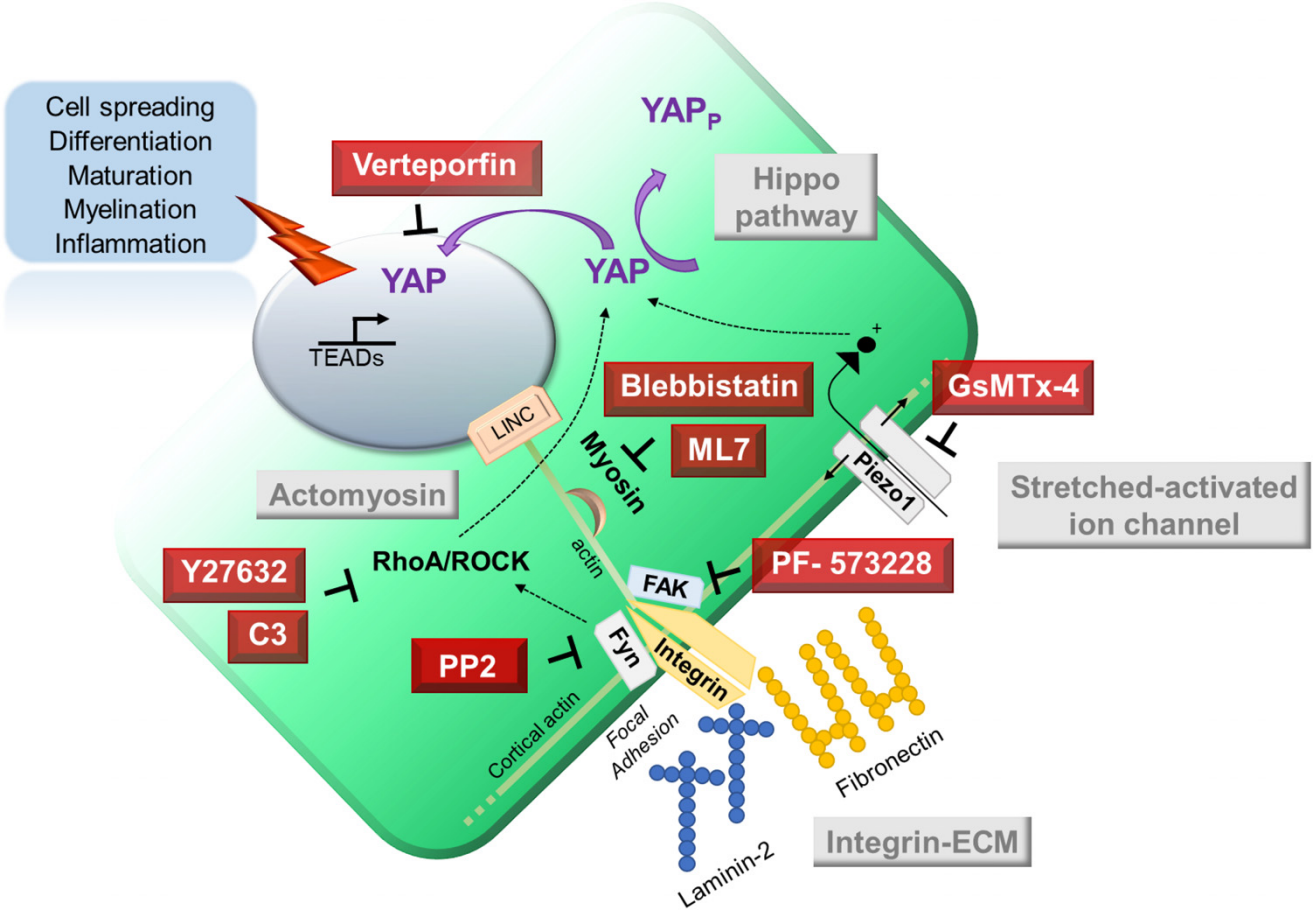
Glial mechanotransduction pathways and pharmacological inhibitors.

Physical changes in the cell microenvironment, including ECM architecture, compression strain, shear stress or osmotic pressure, trigger cell adaptation according to the nature and magnitude of the mechanical signals. The integration, conversion and amplification of these physical signals into biochemical signals are performed by the mechanotransduction process. Hence, mechanosensory systems are distributed over the cell membrane at the interface with the substrate (integrin complexes) or the extracellular fluidic milieu (stretched-activated ion channels). These sensors work closely with the cytoskeletal network (actomyosin), which are in turn connected with adaptor proteins that relay intracellular and nuclear signaling (YAP) and ultimately result in cell architecture changes and morphological adaptation.^58,143^ Cell surface-ligand signaling (integrin-ECM) and the Hippo signaling pathway are well studied although many effectors are still to be confirmed in their sequence of action and partners. Stretched-activated ion channels (Piezo1) are the new kids on the block of glial mechanotransduction. Intraglial variations in preferred pathways have been demonstrated by recent works on Schwan cells^9^ and oligodendrocytes.^10,59^ RNA-seq transcriptome study recently showed that although the Hippo pathways effectors [Large Tumor Suppressor Kinase (LATS), Mammalian Ste20-like Kinase (MST)] are well preserved, YAP and TAZ expression highly varies across the glial cell types.^144^ The identified glial mechanosensors and the underlying mechanotransduction pathways are described in this schematic representation (Fig. 1). Pharmacological inhibitors targeting key molecular actor in mechanotransduction pathway have been used in glial cells, including blebbistatin,^145–147^ verteporfin,^9,112,125^ GsMTX-4,^19,136,148–150^ Y-27632, PP2, PF-573228, C3, and ML-7. The pharmacological inhibitors used to study specific signaling encountered in glia are summarized in Table VI.

**TABLE VI.** Pharmacological inhibitors of cell mechanotransduction used in glia.

Name	Target	References
Blebbistatin	Myosin II	6–10, 46, 47, 54, 57, 63, 119, 145–147, and 59
Verteporfin	YAP-TEAD interaction	9, 112, and 124
	YAP 14–3-3 interaction	
	YAP nuclear translocation	
GsMTX-4	Piezo-1 activity	6, 19, 136, 148, 149, and 150
Y-27632	ROCK	46, 57, 63, 64, 119, 145, 146, and 147
PP2	Src kinases (Fyn)	63, 145, and 122
PF-573228	FAK	63
C3	RhoA/B/C	63
ML-7	Myosin light chain (MLCK)	63 and 119

## V. CURRENT LIMITATIONS

### A. CNS stiffness measurement

In mechanobiology, the brain tissue stiffness is represented by the elastic moduli or the storage Young's modulus, E, and can be measured by indentation through atomic force microscopy (AFM),^18,26,151^ or by using magnetic resonance elastography (MRE).^14^ The latter is noninvasive and often used to assess human brain degeneration and mechanical alteration during aging.^27^ Corrspondingly, the young adult brain displays a storage modulus of approximately 3.5 kPa, while the aged brain possesses lower stiffness ∼2.5 kPa. However, the resolution obtained by MRE is not always defined enough to distinguish atrophic changes in brain geometry and may be subjected to mathematical errors. While AFM measurements are more accurate and, therefore, more widely used in testing animal brain stiffness, both methods must be put in perspective to optimize the comparison between studies.^24^ Murine brain stiffness described in the literature exhibits a value approximately 20 times higher when measured by AFM^36^ compared to the MRE technique.^37^ Regional brain stiffness variation can reach a substantial amplitude. For example, whole rat brain stiffness measured with an indenter set with 25 *μ*m diameter sphere at 1 Hz and 5% strain was found to be between 150 and 300 Pa,^26^ whereas the median stiffness of rat cortical sections was measured in a range between 50 and 500 Pa by using 89.3 *μ*m beads with a force of 20–30 nN.^17^ Therefore, the selection of the tissue sampling method is an important approach to consider when establishing a study model. Particularly, gray and white matters have different permeability and fluid volume content, which modifies their mechanical signature and can alter the stiffness measurement depending on the method employed. For example, CNS sections used for AFM studies do not allow fluid leakage while whole tissue is prone to fluid escape during testing which is one of the main reason thought to explain the differences in brain stiffness measurements.^152^ In addition, the relevance of measuring cerebral stiffness *in vivo* rather than *ex vivo* has just been demonstrated by observing postmortem stiffening of CNS tissues by fluidic and metabolic changes.^153^

Further comparative studies could in future correct the variations in data attributed to specific study methods. Those studies could focus on brain stiffness calibration and direct comparison *in vivo* and *ex vivo* by using MRE and AFM could provide further insights to establish regional tissue maps of the variations in mechanical rigidity within the CNS.

### B. 2D vs 3D engineered methods

Although commonly used, 2D cell culture platforms have several disadvantages when it comes to encompassing physiological forces. To illustrate the lack of natural and physiological representation, the cellular interaction model is greatly restricted by side-by-side linear contact, which sometimes results in cell flattening presenting an altered morphology in contrast to their native behavior. Also, it is noteworthy that cells cultured in 2D will face a poor relevant cell-ECM interaction and that could lead to altered gene expression. Certainly one of the major drawbacks of classic 2D culture systems is the triggered glial immunoreactivity due to dysregulated homeostasis generated by abnormal environment, especially for microglia and astrocytes.^105^ Overall, the use of 2D models should be made for approaches that would target a specific type of interaction to be investigated in order to focus on the expected cellular responses and avoid false positives due to artificially grown culture conditions in order to minimize the risk of having a lack of predictive ability for *in vivo* events. In particular, when myelinating cells are cultured on 2D platforms, the cell body will generate mostly cell processes and spreading but will not achieve an entire membrane wrapping resulting in complete myelination that can be observed around axons.

In contrast, the 3D modeling is particularly attractive because it reproduces more accurately the mechanical, but also structural and geometrical conditions that cells encounter in tissue. The matrix proteins deposited evenly on experimental substrates do not constitute an ideal reproduction of living organisms. Indeed, in humans, matrix fibers exist at many scales of length, which is difficult to model by 2D surface-based substrates. Finally, when a cell applies a tensile force on a compliant substrate attached to stiffer support, the resulting physical deformations are strongly localized and decrease exponentially with the distance from the point of application of the force. The range of deformations is related to the thickness of the substrate.^154^ On the other hand, in the context of a 3D substrate, when a cell contracts on or within the matrix, the deformations extend over relatively long distances and are approximately proportional to the inverse square of the distance from the cell.^132^ In the absence of cross-linking, the tension applied to fiber is all along its length resulting from the nonlinear rheological properties of the matrix.^155–157^ Nevertheless, 3D platforms are not exempted from limitations. At the technical level, 3D culture systems have a higher degree of complexity and thus generate higher costs and a higher demand for expertise and specialized equipment (i.e., bioreactor). Another major problem to consider is the difficulty of visualizing cells in a thick matrix with the usual microscopy techniques. Also, the difficulty to retrieve cells for further downstream analysis can be impaired by the nature and the structure of the system. Laser capture microdissection could be a response to this issue but remains marginally used^125^ due to the difficulty to engineer a suitable system for specific cell collection. Therefore, the matrix features including opacity, biodegradability, pore size or stiffness have to be controlled. In particular, it is common that 3D models are prone to reproducibility defects and exposing great variability within their structure. It is therefore important to consider the most homogeneous distribution possible of the structure to allow an equal distribution of nutrients and biological factors to the cells in order to avoid areas of cellular necrosis.

### C. Glial cell culture model

The observation of cellular phenomena is often carried out over relatively short periods (days or weeks) which could not correlate with the lengthy development of a pathology (months up to years). Although, pathogenesis indeed arises over a long-term period, nonetheless, mechanical changes materialize during a short time. Mechanotransduction is a rapid mechanism translating quick cell behavior change that trigger microenvironmental modifications possibly leading to significant tissue alteration over a longer period. Hence, short-term studies based on single-cell or co-culture are still relevant to unravel specific cell mechanisms but one must remain cautious in extrapolating data toward more complex models of pathologies encompassing diverse topographies, matrix component, cell types and phenotypes.

Glial cell cultures for *in vitro* studies are also limited to the availability of cell type. While sampling mature glia from a living organism is ethically and technically limited, alternatives can be sought in studying organotypic culture on varying mechanical stimuli.^158,159^ The CNS organoid development and the associated malformations can be observed in detail that comes closer to living conditions. However, the level of modeling of mechanical constraints is still sketchy and requires technological advances. Since all of the mechanotransduction platforms without exception have used animal cells, it would be now advisable to consider performing the same experiments with human cells in order to be in line with a development of a therapeutic strategy. Other research would be likely to use gene engineering such as induced pluripotent stem cell (iPSC) technology to induce glial cells-like phenotype from healthy and diseased available patient biopsy (skin or fat mainly). This field is promising and has demonstrated that functional glial cells could be obtained.^160–162^ Human glia could be obtained at any level of commitment or maturation and similar mechanical constraints addressed with animal cells could be eventually assessed. For instance, we can envision that myelination ability of iPSC-derived OLs obtained from healthy and diseases patients (for instance MS) could compared on tunable stiffness artificial axon platform for drug discovery.

## VI. CONCLUSIONS AND PERSPECTIVES

**FIG. 2.**
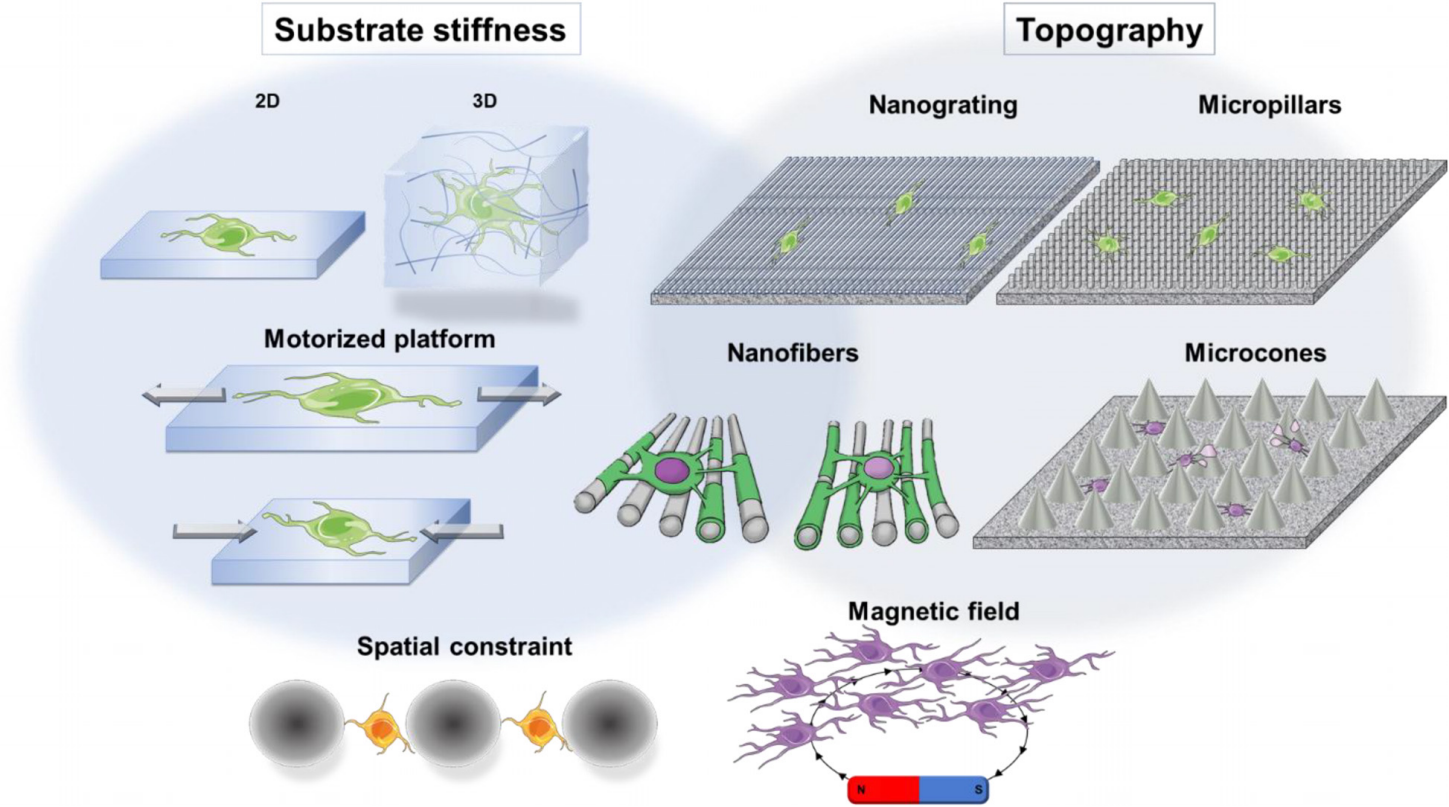
Glial mechanotransduction platforms and assays.

Glial cells are mechanosensitive and their biological responses depend highly on extracellular mechanical features including the nature and stiffness of their substrate, as well as the applied stresses or strains.^38,143^ Mechanical strain in the CNS can arise from several physiological or developmental mechanisms, such as tissue reorganization, fluid flow, and axon growth, as well as pathological events including axon swelling or mechanical trauma. During development, CNS tissue stiffness changes induced by the microenvironment determine the cell morphology and lineage specification. Mechanotransduction studies on cell-substrate interactions can aid design of neuro-glial micro-organs and tissue constructs (Fig. 2). Hence, glial commitment demonstrates sub cell type preferences for substrate stiffness. The mechanisms leading to cell differentiation and maturation are directly influenced by cell shape and morphology. Consequently, the interplay between biochemical and topographical cues is thus driving neuronal and glial cell differentiation. Therefore, the use of testing platforms for drug discoveries promises great advancements in pharmacotherapy of CNS disorders, specifically in the cases of remyelination strategies or astrogliosis prevention. Novel therapeutic targets and biomarkers are expected to be identified following the exploration of these mechanocircuits. As a result, the understanding of the signaling crosstalk between ECM mechanics, fluid flow and mechanosensitive ion channels and their synchronization with paracrine factors to control glial, and more largely, neural cell lineage and behavior would aid in the medicine approaches against neurodegenerative disorders. Therefore, a better understanding of the mechanotransduction pathways involved can potentially identify critical biomolecules for controlling cell fate. Focusing on mechanotransduction signaling to identify specific key molecules as therapeutic target to modulate the glial behavior in disease condition is part of the drug discovery strategy that can arise from those studies. Evidence, which reveals that the effects of drugs acting on the glial system can be influenced by the context of mechanical stiffness established by the disease, is beginning to emerge. By using tunable stiffness platform conjointly with verteporfin treatment, Ong *et al.* highlighted that oligodendrocyte differentiation and maturation may be two mechanisms independently and diversely regulated through the mechanosensory YAP signaling.^125^ Thus, a number of molecules possess a therapeutic potential for the nervous system. Among these molecules, clemastine has shown potential in promoting the differentiation of oligodendrocytes and in aiding the remyelination in defective nervous fibers in preclinical and clinical studies.^163–165^ However, the results are not conclusive over the long term and the pharmacology has yet to be deepened. It is crucial that such molecules with therapeutic potential be tested in models taking into account the mechanical dimensions to evaluate their mode of action and optimize their beneficial activities in order to design a future therapy. In addition, mechanotransduction platforms should also be designed into high throughput devices to help identify new molecules, and this will require manufacturing standardization procedures.

Specifically, no regenerative therapy is effective for the CNS now. In the past decades, several strategies have been used; among them are the cell therapies including the local injection of pluripotent stem cells or mature cells. Nonetheless, cell therapies have often been disappointing due to the poor cell viability. The design of biomaterials to promote healing and regeneration in the nervous system via transplantation of glial progenitors or the implantation of tissue scaffolds is justified.^64^ Biomaterials can physically reproduce the CNS tissue and offer permissive environment for cell survival, growth, and differentiation. Promising results were obtained after the implantation of soft hydrogels at the injury site of CNS tissue which prevent glial scar formation and enabled neurite outgrowth.^131^ However, serious improvements in biomaterial properties are required to extend cell survival and tissue integration. Particularly, the stiffness of the implant can trigger gliosis and inflammation.^166^ Also, preserving a mechanical homeostasis is one of the greatest challenges of the current CNS cell therapy strategies. The knowledge of cell substrate interactions will aid biomaterials design for directing the fate of endogeneous glial cells and exogenous transplanted cells.

## Data Availability

Data sharing is not applicable to this article as no new data were created or analyzed in this study.
